# Post-streptococcal reactive arthritis in children: a distinct entity from acute rheumatic fever

**DOI:** 10.1186/1546-0096-9-32

**Published:** 2011-10-20

**Authors:** Yosef Uziel, Liat Perl, Judith Barash, Philip J Hashkes

**Affiliations:** 1 Pediatric Rheumatology Unit, Pediatric Department, Meir Medical Center, Kfar Saba, Israel and the Sackler School of Medicine, Tel Aviv University, Tel Aviv, Israel; 2 Pediatric Rheumatology Unit, Ambulatory Day Unit, Kaplan Hospital, Rehovot, Hebrew University, Israel; 3 Pediatric Rheumatology Unit, Shaare Zedek Medical Center, Jerusalem, Israel; 4 Cleveland Clinic Lerner School of Medicine of Case Western Reserve University, Cleveland, USA

## Abstract

There is a debate whether post-streptococcal reactive arthritis (PSRA) is a separate entity or a condition on the spectrum of acute rheumatic fever (ARF). We believe that PSRA is a distinct entity and in this paper we review the substantial differences between PSRA and ARF. We show how the demographic, clinical, genetic and treatment characteristics of PSRA differ from ARF. We review diagnostic criteria and regression formulas that attempt to classify patients with PSRA as opposed to ARF. The important implication of these findings may relate to the issue of prophylactic antibiotics after PSRA. However, future trials will be necessary to conclusively answer that question.

## Introductory case 1

An 8 year old boy, previously healthy, presented to the rheumatology clinic with a three week history of right hip pain and one week history of pain in the right knee, without fever. He was diagnosed with streptoccocal tonsilitis one month ago. For his joint complaints he was treated with acetylsalicylic acid for three weeks, with minimal improvement. There was no past personal or family history of arthritis.

On examination, arthritis was found in both the right hip and the right knee. There was no evidence of erythema marginatum, chorea, subcutaneous nodules or cardiac murmurs. The complete blood count was normal, the erythrocyte sedimentation rate (ESR) was 40 mm/hr and antistreptolysin O (ASLO) level was 700 IU/mL. The echocardiogram was normal.

What is the diagnosis? What is the recommended therapy?

## Introductory case 2

A 12 years old girl, previously healthy, presented to the emergency room with 2 days of left knee pain and fever, and was admitted to the pediatric ward for further assessment. She was diagnosed with streptoccocal tonsilitis one month ago. On examination, she had arthritis of the left knee, with no other significant findings. Her complete blood count was normal, the ESR was 90 mm/hr and the ASLO level was 800 IU/mL.

A knee aspirate was completed: synovial fluid had 50,000 white blood cells/mm^3 ^with a predominance of neutrophils. After a day in the hospital, the left knee arthritis disappeared and arthritis of the left hip was detected. She also continued to have fever.

What is the diagnosis? What is the recommended therapy?

## Introductory case 3

A 6 year old girl, previously healthy, presented to the pediatric clinic with a four day history of left knee pain. She had no fever at presentataion, and no history of tonsilitis in the last month. There was no history of trauma. On examination, she had arthritis of the left knee, with no other significant findings. Her complete blood count was normal, the ESR was 60 mm/hr and the ASLO level was 600 IU/mL.

A knee aspirtate was performed: The synovial fluid had 10,000 white blood cells/mm^3 ^with a predominance of monocytes. On follow up there was no involvement of other joints.

What is the diagnosis? What is the recommended therapy?

## Background

### Post infectious arthritis

Post infectious arthritis is defined as arthritis which develops during or soon after an infection elsewhere in the body, but in which the microorganisms cannot be recovered from the joint [1].

The classical pathogens described in association with post infectous arthritis in young children are enteric pathogens: *Salmonella, Shigella, Campylobacter *and *Yersinia*. *Chlamydia trachomatis *is a genital pathogen, which is also known to cause this condition [2]. When these pathogens are involved, the arthritis is termed "Reactive Arthritis" (ReA). Patients with ReA are frequently positive for HLA-B27, and the clinical picture resembles other spondyloarthropathies.

Other infection and post infectious arthritides are caused by viral infections (especially *rubella, mumps, hepatitis B and parvovirus*), *Mycoplasma genitalium, Ureaplasma urealyticum, Chlamydia pneumonia, Neisseria gonorrhea*, and vaccinations with some of the live vaccines. Post infectious arthritis related to β-hemolytic group A streptococcus (GAS) is the focus of this paper.

### Streptococcal post-infectious arthritis

The classic condition related to arthritis following throat infections with GAS is acute rheumatic fever (ARF). The diagnosis of ARF is established largely on clinical grounds. The initial description of the clinical manifestations, now known as the "Jones criteria", was published by Jones in 1944 and revised most lately in 1992. The major criteria (Table 1) include carditis, polyarthritis, chorea, erythema marginatum and subcutaneous nodules. The minor criteria include arthralgia (counted only when arthritis is not present), fever, elevated acute phase reactants and an electrocardiogram showing a prolonged PR interval. If supported by evidence of a preceding GAS infection, the presence of two major manifestations or of one major and two minor manifestations is indicative of a high probability of ARF [3].

**Table 1:** Jones criteria for the diagnosis of acute rheumatic fever (ARF)

	The five major manifestations are
1	Polyarthritis (predominantly involving the large joints)

2	Carditis, valvulitis and pericarditis (eg, pancarditis)

3	Central nervous system involvement (eg, Sydenham chorea)

4	Erythema marginatum

5	Subcutaneous nodules

	**The four minor manifestations are**

1	Arthralgia

2	Fever

3	Elevated acute phase reactants [erythrocyte sedimentation rate (ESR), C-reactive protein (CRP)]

4	Prolonged PR interval

Supporting evidence of antecedent group A streptococcal infection,»

Postive throat culture or rapid streptococcal antigen test

Elevated or rising streptococcal antibody liter

Since 1959, there are reports of patients who present with GAS post infectious arthritis and do not fulfill the classical Jones criteria [4]. This condition is designated as post-streptococcal reactive arthritis (PSRA). The question whether PSRA is a distinct entity from ARF has not yet been fully answered. There are some reports of carditis developing after PSRA, suggesting that PSRA may be part of the spectrum of ARF [5,6]. However, since there are substantial clinical, immunological and genetic differences between PSRA and ARF, we believe PSRA to be a distinct entity [7-9]. This paper will review the entity of PSRA and the major factors distinguishing it from ARF.

### Demographic characteristics of PSRA

The age distribution of PSRA appears to be bimodal; with a peak at ages 8-14 years and another at age 21-37. In contrast, ARF has a single peak incidence in childhood around 12 years, and ReA which has a single peak incidence at 27-34 years [10]. Both genders are equally affected, in all age groups.

### Clinical characteristics of PSRA

#### Disease onset in relation to throat infection

Patients with both PSRA and ARF have arthritis that follow a symptom free interval after an episode of GAS pharyngitis/tonsilitis. In ARF, arthritis usually occurs 10-28 days after the GAS pharyngitis while in PSRA arthritis appears after a shorter "incubation" period, approximately 7-10 days after the infection. Simonini et al. described 52 pediatric PSRA patients, in whom arthritis appeared 4-12 days following pharyngitis [11].

#### Joint involvement (Table 2)

PSRA arthritis is additive and persistent, and can involve large joints, small joints, or the axial skeleton. In ARF the arthritis is migratory and transient, and usually involves the large joints (small and axial joint involvement may occur but is uncommon). In a study by Barash et al. 159 pediatric PSRA patients were compared to 68 ARF patients [12]. Seventy-nine percent of the ARF patients had migratory arthritis compared to 33% of the PSRA patients, and 40% had symmetrical arthritis in the ARF group compared to 22% in the PSRA group. In another series, van Bemmel et al. described 60 adult patients with PSRA [13]. Small joints were involved in 23% of the patients; large joints were involved in 58%, and both types of joints in 18%. Symmetric distribution was found in 60%. Involvement of upper limb joints was found in 18%, lower limb in 50% and both in 32%. Risse et al. described 21 pediatric patients with PSRA of whom 57% had hip arthritis and 43% had knee and/or ankle arthritis; 95% had monoarthritis and 5% oligoarthritis [14]. In all patients the arthritis was not migratory. In the cohort of Simonini et al. monoarthritis involving one large joint was found in 19 children and arthritis involving 2 or 3 joints in 29 [11]. Thirty-seven children had non-migratory arthritis.

**Table 2:** Summary of the character of joint involvement in post-streptococcal reactive arthritis (PSRA)

	*Number of patients*	*Adults/pediatrics*	*% symmetrical*	*% migratory*	*% monoarthritis*	*% oligoarthritis*	*% polyarthritis*
Barash et al. [12]	159	pediatric	22	33	-	-	-
van Bemmel et al. [13]	60	adults	60	-	-	-	-
Risse et al. [14]	21	pediatric	-	0	95	5	0
Simonini et al. [11]	52	pediatric	-	29	36	56	8
Mackie et al. [10]	188	adults & pediatrics	41	18	23	37	37

Mackie et al. [10] conducted a systematic search on Medline using strict inclusion criteria. They identified 188 cases of PSRA published in the literature between 1982-2002, both adult and pediatric. Eighty-two percent had non-migratory arthritis, 23% monoarthritis, 37% oligoarthritis, and 37% polyarthritis. Forty-one percent had symmetrical arthritis. The most frequently involved joints were knee, ankle, wrist, and hip. Nine patients had tenosynovitis.

#### Laboratory markers of inflammation

Barash et al. [12] demonstrated that the ESR and C-reactive protein (CRP) levels were significantly higher in ARF (92.2 mm/h and 10.7 mg/dL, respectively) compared to PSRA patients (57 and 2.3, respectively).

#### Response to treatment and recurrence

The arthritis of ARF responds dramatically to acetylsalicylic acid or NSAIDs like naproxen. In contrast, the response in PSRA is much more modest [7]. Barash et al. reported that the resolution of arthritis after treatment occurred in ARF patients after a mean of 2.2 days compared to 6.9 days in the PSRA group [12]. Relapse occurred in 7% of the ARF group compared with 21% of the PSRA group. van Bemmel et al. described that joint symptoms lasted a mean of 9.7 weeks in his adult PSRA cohort [13]. In the cohort of Risse et al. 33% of PSRA patients continued to have active arthritis after 6 weeks of follow-up [14], while Simonini et al. the reported the mean duration to resolution of symptoms was 54 days [11]. Some patients may benefit from corticosteroid treatment in the acute phase.

### Diagnosis of PSRA

Ayoub et at proposed the following diagnostic criteria [15]:

1. Arthritis of acute onset, symmetric or asymmetric, usually non-migratory, which can affect any joint and is persistent or recurrent. At best, the arthritis is poorly responsive to salicylates or NSAIDs.

2. Evidence of antecedent GAS infection.

3. Failure to fulfill the modified Jones criteria for the diagnosis of ARF.

Recently, Barash et al. suggested a regression mathematical formula based on four significant diagnostic discriminators to differentiate ARF from PSRA [12]:

-1.568 + 0.015 × ESR + 0.02 × CRP - 0.162 × days to resolution of joint symptoms - 2.04 × return of joint symptoms (yes = 1, No = 0)

If the result is greater than 0, the patient is classified as having ARF; otherwise the patient is classified as having PSRA. The sensitivity of this formula was 79% and specificity 87.5% for a correct classification of PSRA.

#### Diagnosis of antecedent Streptococcal infection

In order to diagnose PSRA, evidence of prior GAS infection is necessary. Microbiological confirmation can be obtained by throat culture or rapid antigen detection tests (RADT). However, both throat culture and RADT cannot differentiate a true GAS infection from a carrier state, which can be found in as many as 15% of school age children [7].

Serologic tests are another way of confirmation a recent GAS infection. Elevated or increasing anti-streptococcal antibody titters are of value in identifying a preceding GAS infection in a patient suspected of having PSRA. The most commonly used and commercially available antibody assays are anti-streptolysin O (ASLO) and anti-deoxyribonuclease B (anti-DNase-B).

ASLO titers begin to rise approximately 1 week, and peak 3 to 6 weeks after the initial GAS infection. Anti-DNase-B titers begin to rise 1-2 weeks and peak 6-8 weeks after the infection. Elevated titers for both tests may persist for several months or even years after GAS infection.

A problem in using anti-streptococcal antibody titters in identifying a preceding GAS infection in the pediatric population is that the normal levels of these antibodies are higher among school aged children then among adults [16]. The cutoff level of anti-streptococcal antibody titters that can be considered diagnostic for GAS infection in children is still not clear. Cutoff values of ASLO have ranged from 300-800 IU/ml and 200-800 IU/ml for anti-DNase-B. Some studies have required that titers show a significant longitudinal change. For example, Jansen et al. required a 26% increase in ASLO titers, and 14% in anti-DNase-B titers for inclusion in the study cohort [8,10,14].

It was suggested that levels greater than 2 standard deviations of local laboratory norms, or a two fold increase in the ASLO titer repeated 2-3 weeks after the initial test confirm recent strep infection [8,10,14].

Although GAS is the major pathogen described as causing PSRA other non-group A streptococci (NGAS), including groups C and G, have also been associated with PSRA [10,17]. Jansen et al. proposed differentiating between GAS and NGAS infection in PSRA patients by using an ASLO/anti-DNase-B ratio obtained 4-10 weeks after a throat infection. A ratio less than 1.4 indicates GAS as the cause while a ratio greater than 1.5 suggests NGAS-induced PSRA [18].

### Genetic markers in PSRA

There are several conflicting studies addressing the association of ARF and PSRA with class II HLA-DR antigens. Ahmed et al. found an increased frequency of HLA DRB1*01 in patients with PSRA compared with healthy controls and patients with ARF [9]. In patients with ARF, there was an increased frequency of the HLA DRB1*16 allele when compared with control subjects. This association may suggest that the etiology of PSRA, as of ARF, may be related to the inheritance of certain class II HLA alleles. In contrast Simonini et al. did not find significance differences in frequency of various HLA DRB1 alleles (including DRB1*01 and 16) between 25 patients with ARF, 34 with PSRA and healthy controls [19].

In a study of Israeli patients, Harel et al. [20] has found a significantly higher percentage of B cells expressing the D8/17 antigen in patients with a history of ARF than in control subjects. Later the same group investigated the presence of D8/17 alloantigen on B cells from patients with PSRA compared with control subjects [21]. There was a small but significant difference between the expression of the antigen in patients with PSRA and control subjects, but with significant overlap in the 2 groups. Moreover, there was a weak negative correlation between the percentage of D8/17 positive cells and the time elapsed from diagnosis. Therefore it is not clear whether this alloantigen expression is truly a genetic marker or is induced and regulated by the infection.

### Carditis in PSRA

There are conflicting reports regarding the heart involvement in PSRA. De Cunto et al. described 12 pediatric patients who were diagnosed with PSRA [6]. One of the patients in the group developed classic ARF with valvulitis 18 months after the initial episode. Similarly, Ahmed et al. described 25 pediatric PSRA patients, one of whom developed carditis 9 month from the onset of arthritis [9]. In a retrospective study Moorthy et al. described 40 pediatric patients with PSRA [22]. At baseline, 18% (n = 7) had a finding noted on the echocardiogram such as mild mitral and/or aortic insufficiency, or mitral valve prolapse, 2 patients with a normal baseline echocardiogram may have developed findings after 12 months of follow-up (left ventricular systolic dysfunction, mitral, tricuspid and pulmonary insufficiency). There other case reports and a small series of carditis in PSRA patients [5].

In contrast, JM van Bemmel recently described 60 adult patients diagnosed with PSRA who were not treated with antibiotic prophylaxis [13]. After a median follow up of 8.9 years there was no increased risk of valvular heart disease compared to the control group.

Similarly, Simonini described 52 children with PSRA; all were treated with antibiotic prophylaxis for one year [11]. After a median followup of 8 years none of the patients developed clinical or echocardiographic evidence of valvular disease or cardiac involvement.

Barash et al. [12] described 152 pediatric PSRA patients, none of whom developed carditis on followup [12]. Despite the Jones criteria, discussing only physical findings of carditis as a major diagnostic criteria all children with suspected ARF or PSRA should undergo an echocardiogram as part their work-up.

### Antibiotic prophylaxis in PSRA

In ARF long-term secondary antibiotic prophylaxis is recommended. Thus, the question of secondary prophylaxis arises in PSRA patients. The 2009, American Heart Association (AHA) Scientific Statement recommends that patients with PSRA should be observed carefully for several months for clinical evidence of carditis [7]. They suggest that secondary prophylaxis be given for up to one year after the onset of symptoms and discontinued if there is no evidence of carditis. If valvular disease is detected, the patient should be classified as having had ARF and should continue to receive secondary prophylaxis. However, the effectiveness of this strategy is not well established. The level of evidence (LOE) for this recommendation is C - "only consensus opinion of experts, case studies, or standard of care", and IIb -usefulness/efficacy- less well established by evidence/opinion.

### Back to the cases

#### Case 1

The boy presents with additive arthritis with clinical and serological evidence of a prior streptococcal infection, but does not fulfill the Jones criteria. The probable diagnosis is PSRA. In line with the AHA recommendations, the boy should be evaluated for signs of carditis clinically and by echocardiogram, and treated with prophylactic antibiotic for a year. After a year of treatment the boy should be evaluated again for signs of carditis. If carditis is not observed antibiotic prophylaxis should be discontinued.

#### Case 2

In this case, the girl, at first, represents with fever and monoarthritis. The most important entity to rule out is septic arthritis, and a synovial fluid aspiration must be done, and antibiotic treatment started. Later her arthritis became migratory. Thus she fulfilled the Jones criteria for the diagnosis of ARF with one major (migratory arthritis) and two minor (fever and elevated ESR) criteria. In line with the AHA recommendations, the girl should start long-term secondary antibiotic prophylaxis.

#### Case 3

In this case, the girl presents with reactive arthritis, with no clear evidence of streptococcal infection. The girl did not have clinical tonsillitis, and basing the diagnosis of PSRA on a single ASLO value is problematic. She should be evaluated for clinical and echocardiographic signs of carditis, and ASLO measurement should be repeated in 2-4 weeks. If ASLO titers demonstrate an increase she should be treated as in case 1. If ASLO titers do not increase she likely does not have PSRA and we believe that antibiotic prophylaxis is not justified.

## Conclusions

Current knowledge supports the concept that PSRA is a distinct entity from ARF based on clinical findings, response to therapy and lack of cardiac involvement in almost all cases (Table 3). It is not yet established whether carditis is a late sequela of PSRA and if antibiotic prophylaxis should be given to PSRA patients. With the current low level of evidence supporting prophylaxis in PSRA, further studies in the form of a randomized placebo controlled trial, are required.

**Table 3:** Comparison of post-streptococcal reactive arthritis (PSRA) and acute rheumatic fever (ARF)

	*PSRA*	*ARF*
**Age**	Bimodal: 8-14 years and 21-37 years	5-15 years with peak incidence around 12 years
**Disease onset post streptococcal infection**	7-10 days	10-28 days
**Joint involvment**	Additive and persistent; large, small and axial joints	Migratory, transient; mainly large joints
**Acute phase reactants**	Moderatly elevated	Markedly elevated
**Response of arthritis to acetylsalicylic acid or NSAID treatment**	Poor to moderate	Dramatic
**Genetic markers**	Increased frequency of HLA DRB1*01	Increased frequency of the HLA DRB1*16 allele
**Carditis**	Conflicting reports, but uncommon	Major diagnostic criteria, between 60-70%
**Antibiotic prophylaxis**	Antibiotic prophylaxis for one year if echocardiogram is normal	Long-term secondary antibiotic prophylaxis

NSAID: Non-steroidal antiinflammatory drugs

## List of Abbreviations

ASLO: anti streptolysin O; ARF: acute rheumatic fever; PSRA: post-streptococcal; reactive arthritis; ReA: reactive arthritis; GAS: Group A β hemolytic streptococcus; CRP: C-reactive protein; RADT: rapid antigen detection tests; DNase-B: deoxyribonuclease B; AHA: American Heart Association.

## Competing interests

The authors declare that they have no competing interests.

## Authors' contributions

YU participated in literature review and writing the manuscript. LP participated in literature review and writing the manuscript. JB participated in literature review and writing the manuscript. PJH participated in literature review and writing the manuscript. All authors read and approved the final manuscript.
